# Pharmacological Effects of Angiotensin 1–7 on Venous Vascular Tone

**DOI:** 10.3390/biomedicines14051056

**Published:** 2026-05-07

**Authors:** Armond Daci, Hygerta Berisha, Era Rexhbeqaj, Ilir Berisha, Kaltrina Zenuni, Paolo Magni

**Affiliations:** 1 Department of Pharmacy, Faculty of Medicine, University of Prishtina, 10000 Prishtina, Kosovo; hygerta.berisha@unimi.it (H.B.); era.rexhbeqaj@student.uni-pr.edu (E.R.); 2 Department of Pharmacological and Biomolecular Sciences “Rodolfo Paoletti”, Universita Degli Studi di Milano, Via Balzaretti 9, 20133 Milan, Italy; paolo.magni@unimi.it; 3 Faculty of Medicine, University of Prishtina, 10000 Prishtina, Kosovo; ilir.berisha13@student.uni-pr.edu; 4 Faculty of Pharmacy, Alma Mater Europaea Campus Kolegji “Rezonanca”, 10000 Prishtina, Kosovo; kaltrina.zenuni@rezonanca-rks.com

**Keywords:** angiotensin (1–7), MAS receptor, RAAS, vascular tone, vasodilation, K^+^ channels, inferior vena cava, cardiovascular regulation

## Abstract

**Background/Objectives**: The ACE2/Ang-(1–7)/Mas receptor axis is a protective, counter-regulatory component of the RAAS that opposes Ang II/AT_1_R-mediated vasoconstriction. The present study evaluated the pharmacological effects of Ang-(1–7) in the rat inferior vena cava (IVC), a venous capacitance vessel involved in the regulation of venous return and cardiac preload. We hypothesized that Ang-(1–7) exerts anti-contractile effects in the rat inferior vena cava through activation of potassium channel-dependent mechanisms in venous smooth muscle. **Methods**: Isolated IVC rings from Wistar rats were studied using organ bath assays. Ang-(1–7) effects were assessed on pre-constriction induced by angiotensin II (Ang II), phenylephrine (PE), endothelin-1 (ET-1), and thromboxane A_2_ analog (U46619). Responses were recorded and quantified. Mechanistic involvement of nitric oxide (NO), prostaglandins, soluble guanylate cyclase (sGC), and K^+^ channels was evaluated using specific pharmacological inhibitors. **Results**: Ang-(1–7) attenuated Ang II-induced contraction. The effect was markedly reduced by tetraethylammonium (TEA), indicating a predominant role of potassium channel-dependent mechanisms in venous smooth muscle. In contrast, inhibition of nitric oxide synthase, soluble guanylate cyclase, or cyclooxygenase had minimal influence. Ang-(1–7) also produced concentration-dependent relaxation in PE-, ET-1-, and U46619-precontracted vessels, demonstrating agonist-dependent anti-contractile activity. **Conclusions**: Ang-(1–7) exerts significant anti-contractile effects in the rat inferior vena cava primarily through activation of TEA-sensitive K^+^ channels in venous smooth muscle. These findings demonstrate functional activity of the ACE2/Ang-(1–7)/Mas axis in a major venous capacitance vessel and provide mechanistic insight into Ang-(1–7)-mediated modulation of venous tone, supporting further investigation in in vivo models.

## 1. Introduction

The renin–angiotensin–aldosterone system (RAAS) plays a fundamental role in the regulation of vascular tone, electrolyte balance, and blood pressure, and its dysregulation contributes to the development of hypertension, atherosclerosis, and other cardiovascular diseases [1]. Traditionally, the biological effects of the Renin–Angiotensin–Aldosterone System (RAAS) have been attributed to the classical Angiotensin-Converting Enzyme (ACE)/Angiotensin II (Ang II)/AT_1_ receptor axis, which promotes vasoconstriction, inflammation, and vascular remodeling [2]. However, increasing evidence identifies an alternative counter-regulatory pathway, the ACE2/Ang-(1–7)/Mas receptor axis, which mediates vasoprotective, anti-inflammatory, and antifibrotic effects and opposes many of the deleterious actions of Ang II [3,4]. Ang-(1–7) is a heptapeptide generated mainly from Ang II or Ang I through the catalytic activity of ACE2. The binding of Ang-(1–7) to the Mas receptor, a G protein-coupled receptor, generally produces effects opposite to those of Ang II. Activation of this pathway promotes vasodilation, enhances nitric oxide (NO) and prostacyclin release, and modulates vascular smooth muscle tone [5,6]. Beyond endothelial mechanisms, Ang-(1–7) has been shown to directly regulate vascular smooth muscle excitability through activation of potassium channels and modulation of intracellular signaling pathways, including ERK/MAPK and RhoA/Rho-kinase cascades [7,8,9]. These effects are associated with antihypertensive and cardioprotective outcomes, suggesting that Ang-(1–7) represents a promising therapeutic target for cardiovascular disorders [10,11]. Consequently, pharmacological strategies aimed at enhancing ACE2 activity, administering stable Ang-(1–7) analogs, or developing Mas receptor agonists have emerged as potential next-generation approaches complementing conventional ACE inhibitors (ACEi) and angiotensin receptor blockers (ARBs) [6,12,13]. Although the vascular actions of Ang (1–7) have been extensively investigated in various arterial tissues, such as the aorta, mesenteric arteries, and coronary vessels [14,15], its functional role in venous vascular regulation remains poorly characterized. Large capacitance veins, such as the inferior vena cava, differ fundamentally from resistance arteries in ion channel expression, Ca^2+^ sensitization dependence, and their primary hemodynamic function in regulating stressed blood volume and cardiac preload [16]. Consequently, mechanistic extrapolation from arterial studies cannot adequately define venous responsiveness. Importantly, large veins such as the inferior vena cava (IVC) represent the major capacitance vessels of the circulation and play a critical role in controlling venous return, cardiac preload, and overall systemic hemodynamics. Unlike arteries, veins exhibit greater compliance, distinct ion channel regulation, and enhanced reliance on Ca^2+^ sensitization mechanisms [17,18], making extrapolation from arterial pharmacology to venous physiology inadequate. Therefore, direct mechanistic investigation of venous smooth muscle responses is required rather than extrapolation from arterial pharmacology.

To our knowledge, no previous study has systematically characterized the concentration–response relationship and mechanistic pathways of Ang-(1–7) in isolated rat IVC. This represents a significant gap in RAAS research, particularly given the increasing recognition that venous tone contributes to blood pressure regulation, cardiac preload, and congestion in cardiovascular disease [13,19,20]. Previous studies have shown that experimental inhibition of the endothelin-1 receptor in thoracic IVC constriction (TIVCC) reduces the systemic pressure caused by RAAS activation [21]. In addition, pharmacological targeting of the RAAS pathway has been shown to reduce experimental venous thrombosis [22]. These findings underscore the importance of venous-targeted RAAS modulation beyond traditional arterial-focused approaches. Importantly, emerging in vivo evidence supports a biologically relevant role of Ang-(1–7) within the venous circulation. Experimental studies demonstrate that Ang-(1–7) attenuates venous thrombosis [23]. In addition, Ang-(1–7) has been shown to reduce neointimal hyperplasia in venous graft models [24]. Moreover, activation of the alternate RAS axis, including Ang-(1–7)/Mas, has also been implicated in portal hypertension and splanchnic vascular modulation in animal studies and reviews [25]. However, these investigations did not provide detailed concentration–response analysis or pharmacological inhibitor profiling in isolated large capacitance veins. Therefore, a systematic mechanistic characterization of the inferior vena cava remains necessary.

Recent advances in cardiovascular pharmacology emphasize the need to explore mechanisms that may complement or improve upon conventional RAAS inhibition. While ACEi and ARBs primarily attenuate Ang II signaling, enhancement of the ACE2/Ang-(1–7)/Mas axis may provide additive vasoprotective effects with potentially distinct hemodynamic profiles [6,12,26].

Although Ang-(1–7)-induced vasorelaxation has been characterized in arterial vessels, systematic mechanistic investigation in large venous capacitance vessels remains limited. In particular, the relative contribution of K^+^ channels, nitric oxide, prostaglandins, and cGMP-dependent pathways in venous smooth muscle remains undefined. By using multiple constrictors and examining K^+^ channel-dependent mechanisms, we sought to clarify the agonist-dependent anti-contractile effects of Ang-(1–7) and its potential contribution to venous regulation and cardiovascular homeostasis.

## 2. Materials and Methods

### 2.1. Study Design, Setting, and Ethical Approval

This study was designed as an experimental in vitro investigation to evaluate the effects of Ang (1–7) on vascular tone using isolated segments of the IVC from laboratory rats. All experiments were performed at the Laboratory of Experimental Medicine, Faculty of Medicine, University of Prishtina, under controlled conditions and following internationally accepted guidelines for good laboratory practice.

All procedures involving animals were conducted in accordance with the ethical principles for the care and use of laboratory animals. The study protocol was reviewed and approved by the Ethics Committee of the Faculty of Medicine, University of Prishtina (Approval No. 2338).

### 2.2. Experimental Animals and Maintenance Conditions

A total of fifteen adult male Wistar rats (weighing 150–250 g) were used in this research. The animals were housed in standard laboratory cages (DGM™ ventilated animal research cages, Tecniplast, Buguggiate, Italy) with constant environmental parameters (temperature 22 ± 2 °C, humidity 50 ± 10%, and a 12-h light/dark cycle) and had free access to standard chow and water.

All animals were acclimatized for at least one week before the experiments. Euthanasia was performed by carbon dioxide (CO_2_) inhalation, minimizing animal distress and ensuring a humane sacrifice, in line with institutional and international ethical standards.

### 2.3. Isolation and Preparation of IVC Segments

Immediately after euthanasia, the abdominal cavity was carefully opened through a midline incision. IVC was identified, dissected free from surrounding connective and adipose tissues, and immediately immersed in ice-cold oxygenated Krebs solution.

The Krebs solution was freshly prepared in the laboratory with the following composition (in mM): NaCl 118.0, KCl 4.7, CaCl_2_ 2.5, KH_2_PO_4_ 1.2, MgSO_4_ 1.2, NaHCO_3_ 25.0, and glucose 11.0 (all reagents from Sigma-Aldrich, St. Louis, MO, USA). The glucose concentration (11 mM) corresponds to the standard Krebs–Henseleit solution used in vascular organ bath experiments to maintain tissue viability and was not intended to model hyperglycemia. The solution was continuously oxygenated, and pH was maintained at 7.4. The excised venous tissue was gently cleaned and cut into 3–4 mm long rings, which were subsequently mounted for functional testing in organ bath chambers.

### 2.4. Bath Setup and Tissue Equilibration Procedure

Each venous ring was mounted horizontally between two stainless steel hooks in 10 mL organ bath chambers filled with Krebs solution maintained at 38 °C and continuously aerated with a 95% O_2_/5% CO_2_ gas mixture to sustain physiological oxygenation and pH. The preparations were subjected to an initial resting tension of 0.6 g and allowed to equilibrate for 60 min, during which the bath solution was refreshed every 15–20 min. This equilibration ensured tissue stability and consistent contractile responsiveness before the initiation of experimental protocols.

### 2.5. Experimental Protocols and Application of Pharmacological Agents

**Figure 1:**
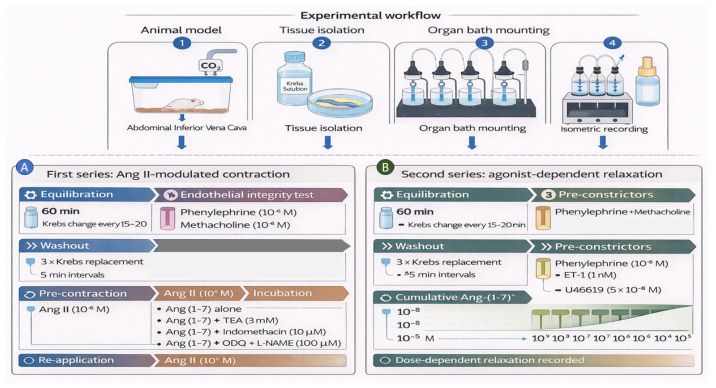
Experimental workflow and pharmacological protocols used in isolated rat inferior vena cava preparations. (**A**) After tissue isolation and equilibration, venous rings were precontracted with phenylephrine, followed by methacholine-induced relaxation to verify intact endothelial function. In the first experimental series, Ang II-induced contraction was recorded, after which tissues were incubated with Ang-(1–7) alone or in the presence of pharmacological inhibitors (TEA, indomethacin, or ODQ + L-NAME). Ang II was then re-applied to assess the modulatory effect of Ang-(1–7). (**B**) In separate experiments, after endothelial assessment, venous rings were precontracted with phenylephrine, endothelin-1, or U46619, followed by cumulative application of Ang-(1–7) (10^−9^–10^−5^ M) to evaluate concentration-dependent relaxation.

**Figure 2:**
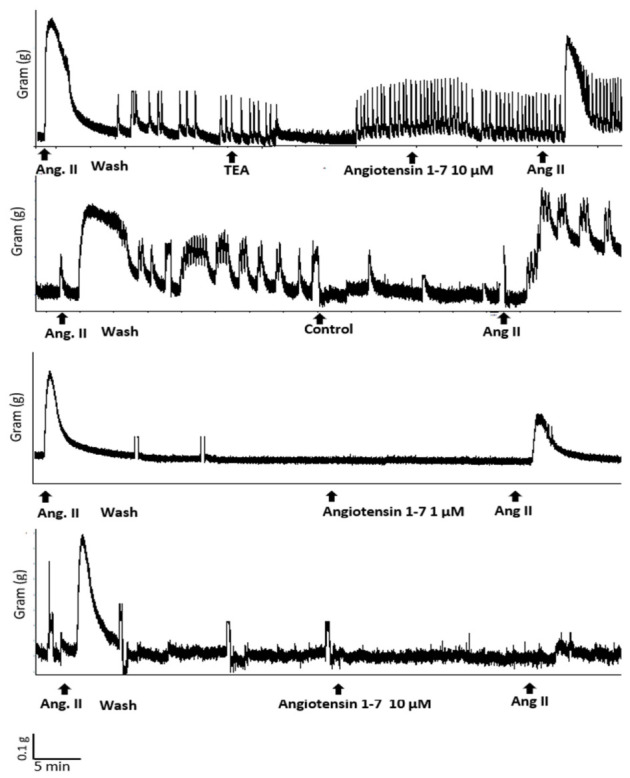
Comparison of representative experimental traces showing the inhibitory effect of Ang-(1–7) on Ang II-induced contractions in inferior vena cava (IVC) rings before and after incubation with Ang-(1–7), and following inhibition with TEA.

After stabilization, the functional responses of the venous rings were assessed using a series of vasoconstrictor and vasodilator agents. The overall experimental workflow and pharmacological protocols used in this study are summarized schematically in Figure 1. Following the 60 min equilibration period described above, endothelial integrity was assessed before initiation of pharmacological experiments. Rings were first precontracted with phenylephrine (10^−5^ M; Sigma-Aldrich, St. Louis, MO, USA), after which methacholine (10^−5^ M; Sigma-Aldrich, St. Louis, MO, USA) was added to evaluate endothelium-dependent relaxation. Preparations exhibiting >70% relaxation of the phenylephrine-precontracted tone were considered endothelium-intact and were included in the experimental series. This endothelial integrity test was performed once at the beginning of the first experimental series. In the first series of experiments, we examined the ability of Ang-(1–7) to modulate Ang II-induced contractile responses in isolated inferior vena cava (IVC) rings. After an equilibration period, Ang II (10^−6^ M; Sigma-Aldrich, St. Louis, MO, USA) was added to the organ bath to induce a stable contraction. The contractile response was allowed to develop for approximately 5–10 min until a steady plateau was reached. Following the development of a sustained response, tissues were washed three times with fresh Krebs solution and allowed to re-equilibrate. Washout consisted of three complete bath solution replacements performed at approximately 5 min intervals, after which tissues were allowed to stabilize for an additional 10–15 min before the next intervention. The same venous rings were then incubated with Ang-(1–7) (10^−5^ M; Sigma-Aldrich, St. Louis, MO, USA) for 15–20 min. After incubation, Ang II (10^−6^ M) was re-applied, and the resulting contractile response was recorded to evaluate the modulatory effect of Ang-(1–7). To investigate the mechanisms underlying the modulatory effect of Ang-(1–7), additional experiments were performed in the presence of pharmacological inhibitors. After washout and re-equilibration, IVC rings were pre-incubated for 10–15 min with the respective inhibitor before Ang-(1–7) administration: tetraethylammonium (TEA, 3 mM; Sigma-Aldrich, St. Louis, MO, USA), a combination of endothelial nitric oxide synthase (eNOS) inhibitor L-NAME (100 µM; Sigma-Aldrich, St. Louis, MO, USA) and a soluble guanylate cyclase (cGMP pathway) inhibitor ODQ (10 µM; Sigma-Aldrich, St. Louis, MO, USA), or prostaglandin synthesis inhibitor indomethacin (10 µM; Sigma-Aldrich, St. Louis, MO, USA). Ang-(1–7) (10^−5^ M) was then added in the continued presence of the inhibitor, followed by re-application of Ang II (10^−6^ M). These inhibitors were selected to distinguish endothelial-dependent mechanisms from direct smooth muscle pathways and to identify the predominant signaling mechanism involved in Ang-(1–7)-mediated venous relaxation. Contractile responses were recorded and compared with control experiments performed in the absence of inhibitors. Angiotensin II was unsuitable for direct relaxation experiments because its contractile response was transient and rapidly decayed rather than reaching a sustained plateau (see original traces, Figure 2). This behavior, consistent with AT_1_ receptor tachyphylaxis and internalization, prevents reliable assessment of direct vasorelaxant responses. To induce vascular contraction, the following agonists were added to the organ baths at the indicated concentrations: PE (10^−5^ M), an α_1_-adrenergic receptor agonist; ET-1 (1 nM; Sigma-Aldrich, St. Louis, MO, USA), a potent endothelial-derived vasoconstrictor; Thromboxane A_2_ analog U46619 (5 × 10^−8^ M; Cayman Chemical, USA), a stimulator of TP receptors; and Ang II (10^−5^ M), a classical RAAS component promoting vasoconstriction. To confirm endothelial integrity, methacholine (10^−5^ M) was applied in preparations, eliciting endothelium-dependent relaxation. Following the establishment of a stable pre-constriction with one of the agents above, Angiotensin Fragment 1–7 (acetate)—Ang (1–7) (10^−9^–10^−5^ M; Cayman Chemical, USA)—was administered cumulatively to assess its vasodilatory effect mediated through MAS receptor activation. All pharmacological substances were freshly prepared in Krebs solution immediately before use to ensure reproducibility and chemical stability.

### 2.6. Recording of Isometric Tension and Data Collection

Changes in isometric tension were continuously recorded using force transducers connected to a LabChart data acquisition system (ADInstruments, Dunedin, New Zealand). The contractile response produced by each vasoconstrictor was considered the baseline (100% contraction), and subsequent relaxations induced by Ang (1–7) were expressed as a percentage of the initial contraction. All recordings were saved and analyzed offline.

### 2.7. Statistical Analysis

Graphical and numerical data analyses were performed using GraphPad Prism version 9.0 (GraphPad Software, San Diego, CA, USA). Data are presented as mean ± standard error of the mean (SEM) (*n* = 8). Contractile responses were normalized to the initial precontraction and expressed as a percentage (%) of the maximal contractile response (E_max_). For comparisons involving multiple treatment groups with a single independent factor, statistical significance was evaluated using one-way analysis of variance (ANOVA) followed by Tukey’s multiple comparisons post hoc test. For experiments involving two independent variables, statistical significance was assessed using two-way ANOVA followed by Bonferroni’s post hoc test. Comparisons between two groups were performed using an unpaired Student’s *t*-test, where appropriate. A *p*-value < 0.05 was considered statistically significant. Specifically, * *p* < 0.05 and *** *p* < 0.001 denote significance versus Ang II alone.

## 3. Results

### 3.1. Effect of Ang-(1–7) on Ang II-Induced Contraction

**Figure 3:**
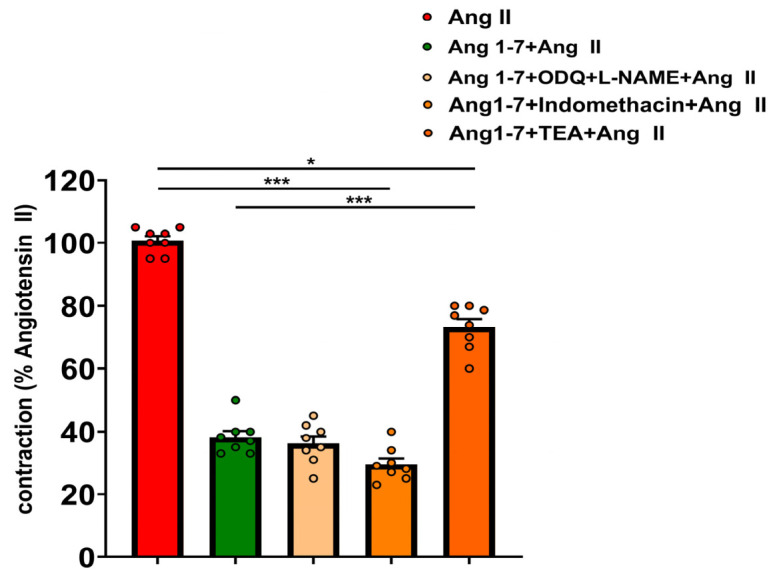
Effect of Ang-(1–7) pretreatment on Ang II-induced reactivity of isolated IVC rings in vitro and the influence of pharmacological inhibitors. Vascular responses to Ang II were compared between Ang-(1–7)-pretreated and non-treated IVC rings in the absence or presence of L-NAME (eNOS inhibitor), the combination of L-NAME + ODQ (cGMP pathway inhibition), TEA (K^+^ channel inhibitor), or indomethacin (cyclooxygenase inhibitor). Data are expressed as mean ± SEM of maximal response (E_max_, % of precontraction) (*n* = 8). Direct comparison between Ang-(1–7) + Ang II and Ang-(1–7) + TEA + Ang II was performed to evaluate the contribution of potassium channels. Statistical analysis was performed using one-way ANOVA followed by Tukey’s multiple comparisons test * *p* < 0.05, and *** *p* < 0.001 indicate statistical significance versus Ang II alone.

In the first series of experiments, pretreatment with Ang-(1–7) significantly reduced the contractile response to Ang II in isolated inferior vena cava rings. Following incubation with Ang-(1–7), the Ang II-induced contraction was markedly attenuated compared with the initial response (Ang II 10^−6^ M: E_max_ = 100.750 ± 2.67% vs. Ang-(1–7) 10^−5^ M + Ang II 10^−6^ M: E_max_ = 41.14 ± 2.67%; *p* < 0.0001). This inhibitory effect of Ang-(1–7) was significantly attenuated in the presence of the potassium channel blocker TEA. Pretreatment with TEA markedly increased the Ang II-induced contractile response compared with Ang-(1–7) alone (Ang-(1–7)+Ang II vs. Ang-(1–7) + TEA + Ang II), with an E_max_ of 73.5 ± 4.5%, indicating substantial reversal of the Ang-(1–7)-mediated inhibition. Represented original traces are shown in Figure 2, and the corresponding quantitative analysis is presented in Figure 3. The strong attenuation of the Ang-(1–7) effect by TEA supports a dominant role of potassium channel activation in mediating venous smooth muscle hyperpolarization. In contrast, the absence of significant effects of L-NAME and ODQ or indomethacin suggests that nitric oxide and prostaglandin pathways do not represent the primary contributors in this venous preparation (Figure 3). To further evaluate its inhibitory effect, additional experiments were performed using lower Ang-(1–7) concentrations (10^−7^ M and 10^−6^ M), from which the IC_50_ of Ang-(1–7) for inhibiting Ang II-induced contraction was estimated to be ≈2.14 × 10^−6^ M (Ang II E_max_ = 101.29 ± 2.67% vs. Ang-(1–7) 10^−6^ M + Ang II 10^−6^ M E_max_ = 55.34 ± 2.47%, *p* < 0.0001). Overall, Ang-(1–7) inhibited contractile responses, confirming inhibition against Ang II-induced constriction. These findings highlight the functional role of the Ang-(1–7)/Mas receptor axis in modulating vascular reactivity and counterbalancing the classical renin–angiotensin–aldosterone system (RAAS) pathway.

### 3.2. Vasorelaxant Effect of Ang (1–7) on IVC Rings Pre-Constricted with PE, ET-1, and U46619

**Figure 4:**
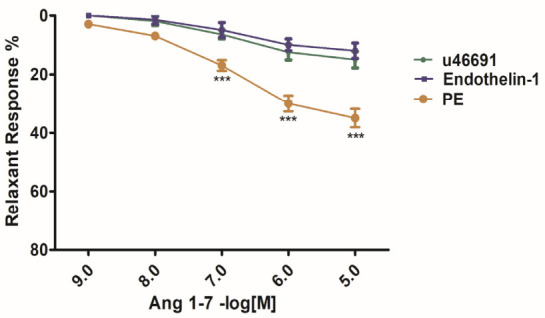
Concentration-dependent direct relaxation responses of IVC rings to Ang-(1–7) precontracted with different constrictors. Vessels were precontracted with phenylephrine (PE), U46619 (thromboxane A_2_ analog), or endothelin-1 (ET-1). Relaxation responses were measured in three experimental groups over a concentration range from 1 nM (10^−9^ M) to 10 µM (10^−5^ M). The first point (0) represents the baseline prior to Ang-(1–7) administration. Data are presented as mean ± SEM of maximal relaxation response (*n* = 8). Relaxation responses are expressed as a percentage (%) of the respective precontraction level (E_max_). Statistical significance was evaluated using two-way analysis of variance (ANOVA), followed by a post hoc test of Bonferroni. *** *p* < 0.001 indicates statistical significance between PE versus ET-1 and U46619. Time-control experiments performed in parallel confirmed overall stability of agonist-induced tone, with only minor, non-significant decline observed in phenylephrine-precontracted rings.

In the second round of experiments, the dose-dependent vasorelaxant effect of Ang (1–7) was evaluated in IVC rings that were pre-constricted with more stable constrictors ET-1 (1 nM), PE (10^−5^ M) and U46619 (30 nM) before cumulative administration of Ang (1–7) to the organ bath at 5 min intervals for each dosage. Phenylephrine (10^−5^ M) produced a stable precontraction of approximately 0.7–0.9 g, while ET-1 (1 nM) and U46619 (30 nM) were used at concentrations selected to produce pharmacologically comparable levels of contractile tone across preparations. In all preparations, Ang (1–7) produced a concentration-dependent relaxation of PE, ET-1, and u46619-induced contractions. The relaxation response was most evident at a higher dosage, as represented in Figure 4 and Table 1.

Overall, these findings suggest that Ang (1–7) can attenuate PE, ET-1-mediated, and u46619-mediated venoconstriction in a concentration-dependent manner, supporting its role in the modulation of vascular tone. Ang-(1–7) induced the largest relaxation in PE-precontracted rings, intermediate relaxation in ET-1, and the smallest relaxation in U46619-precontracted rings. These differences reflect the relative potency and receptor-mediated signaling of the constrictors, with PE contractions being more sensitive to Ang-(1–7)-mediated endothelium-dependent relaxation, while U46619 and ET-1 produce more potent and sustained contractions that are less susceptible to this pathway. Overall, Ang (1–7) produced a moderate vasodilatory effect for PE (Table 1), but less pronounced than the effect for ET-1 and u46619. The relaxation responses appeared to be concentration-dependent but of lower magnitude, suggesting that the ability of Ang (1–7) to counteract thromboxane-induced and ET-1-induced constriction may be limited or mediated by different receptor mechanisms. Time-control experiments performed in the absence of Ang-(1–7) demonstrated no significant decline in agonist-induced tone over the experimental period. Although a slight reduction in tone was observed in phenylephrine-precontracted rings, this was not statistically significant and did not affect the interpretation of relaxation responses. Overall, these findings demonstrate that while Ang-(1–7) exerts detectable relaxation in precontracted venous rings, there are differences in Ang-(1–7) activity depending on the preconstricting agonist.

## 4. Discussion

The present experimental study investigated the pharmacological effects of Ang-(1–7) in the rat inferior vena cava (IVC), with particular emphasis on its anti-contractile properties and underlying mechanisms. Consistent with a functional venous role of the ACE2/Ang-(1–7)/Mas axis, previous in vivo studies have demonstrated that Ang-(1–7) reduces experimental venous thrombosis and attenuates venous graft remodeling [23]. These observations suggest that Ang-(1–7) contributes to venous protection under pathological conditions. However, prior investigations did not directly dissect concentration–response relationships or identify the dominant signaling mechanisms in isolated large capacitance veins. The present study, therefore, complements existing in vivo data, providing pharmacological evidence suggesting involvement of potassium channel-dependent mechanisms underlying Ang-(1–7)-mediated venous modulation. Our findings demonstrate that Ang-(1–7) significantly attenuates Ang II-induced contraction in the rat IVC, thereby extending the functional relevance of the ACE2/Ang-(1–7)/Mas receptor axis to the venous circulation and providing direct experimental evidence that this protective RAAS arm operates in capacitance vessels rather than being extrapolated from arterial observations. A principal finding of this study is the potent inhibition of Ang II-evoked venoconstriction by Ang-(1–7). Similar antagonistic interactions between Ang-(1–7) and Ang II have been reported in arterial beds [9,19,27]. The anti-contractile effect in the IVC was significantly attenuated by the broad potassium channel blocker TEA, suggesting that activation of TEA-sensitive K^+^ channels contributes critically to the Ang-(1–7) response. The marked attenuation of the Ang-(1–7) response by TEA, together with the absence of significant effects of L-NAME, ODQ, or indomethacin, indicates that potassium channel-dependent hyperpolarization represents a dominant mechanism underlying Ang-(1–7)-mediated modulation of venous tone. Activation of potassium channels by Ang-(1–7) represents a critical downstream mechanism underlying venous relaxation. Opening of potassium channels promotes membrane hyperpolarization in vascular smooth muscle cells, which reduces the activation of voltage-dependent Ca^2+^ channels and leads to a decrease in intracellular Ca^2+^ concentration. This reduction in Ca^2+^ availability limits actin–myosin interaction and promotes smooth muscle relaxation. In vascular smooth muscle, Ang-(1–7) has been shown to enhance potassium currents and induce hyperpolarization, supporting a K^+^ channel-dependent mechanism of vasorelaxation [20]. While the specific potassium channel subtype cannot be definitively identified under the current experimental conditions, these findings are consistent with the known contribution of large-conductance Ca^2+^-activated K^+^ (BKCa) channels in regulating vascular tone. Furthermore, the ACE2/Ang-(1–7)/Mas axis is recognized as a key regulator of vascular function, capable of modulating ion channel activity, including potassium channel-dependent regulation of membrane potential and intracellular signaling pathways [11,14]. The magnitude of TEA-mediated attenuation suggests that potassium channel activation contributes substantially to the Ang-(1–7) response in venous smooth muscle under our experimental conditions, distinguishing the venous response profile from predominantly nitric-oxide-dependent arterial preparations in the literature. This finding underscores the importance of direct venous investigation rather than arterial extrapolation. These findings indicate that smooth muscle hyperpolarization mediated through TEA-sensitive K^+^ channels likely contributes to the observed response, supporting a predominantly smooth muscle-centered mechanism in this venous preparation. These observations are consistent with Haulică et al. (2003), who reported that Ang-(1–7) reduces Ang II-induced contraction in rat aortic rings even in endothelium-denuded vessels, indicating a direct smooth muscle effect [19].

Ang-(1–7) also functions as a counter-regulatory component of the renin–angiotensin–aldosterone system (RAAS). It can oppose Ang II through AT_1_ receptor modulation, intracellular signaling pathways, and local RAS inhibition. Although Mas receptor antagonism was not tested here, previous studies show that Ang-(1–7) can attenuate Ang II-induced contraction even when Mas is blocked [19,28]. This raises the possibility of Mas-independent or AT_2_ receptor-mediated signaling pathways, which have been described in certain vascular contexts and may contribute to venous responses. Importantly, the lack of effect of nitric oxide inhibitors indicates that NO–cGMP signaling does not contribute significantly to the Ang-(1–7) response in the present preparation. Because AT_2_ and several Mrg-related receptor pathways are typically associated with NO-dependent vasorelaxation, these mechanisms appear less likely to account for the observed effect. Instead, the strong sensitivity to TEA suggests a potassium channel-dependent mechanism consistent with smooth muscle hyperpolarization, although confirmation will require receptor-selective antagonists. However, receptor-specific contributions in venous tissue remain to be clarified and require further investigation using selective antagonists or genetic approaches. Additional intracellular mechanisms described in the literature include modulation of ERK/MAPK, p38 MAPK, Gq/PLC/IP_3_ signaling, and inhibition of RhoA/Rho-kinase-mediated Ca^2+^ sensitization [8,9] Given that venous smooth muscle exhibits enhanced reliance on Ca^2+^ sensitization mechanisms compared with arteries, inhibition of RhoA/Rho-kinase signaling may represent an additional complementary pathway through which Ang-(1–7) reduces venous tone.

Venous smooth muscle exhibits functional differences from arterial smooth muscle, including greater reliance on Ca^2+^ sensitization and distinct ion channel regulation [29]. Large veins such as the IVC regulate venous return and cardiac preload; thus, Ang-(1–7)-mediated modulation of venous tone may influence systemic hemodynamics. Importantly, extrapolation from arterial data to venous physiology cannot be assumed, as veins primarily regulate vascular capacitance and stressed blood volume rather than peripheral resistance. Moreover, veins often display stronger contractile responses to vasoactive agents such as Ang II, endothelin-1, and thromboxane analogs compared with arteries of similar caliber, underscoring the importance of direct venous investigation. The present data therefore provide direct experimental evidence that the protective ACE2/Ang-(1–7)/Mas axis operates functionally within venous capacitance vessels rather than being inferred solely from arterial observations. In our experiments, Ang-(1–7) exhibited agonist-dependent relaxations, with the strongest effects against PE-induced contraction and weaker effects against ET-1 and U46619. This indicates selective modulation of specific venous contractile pathways rather than uniform Ang II antagonism. The weakest relaxations were observed with ET-1 and U46619, consistent with arterial data where thromboxane-mediated pathways are less influenced by Mas receptor signaling [18,30,31]. This agonist-dependent variability suggests that Ang-(1–7) preferentially interferes with Ang II-linked signaling cascades, while exerting more modest modulation of endothelin- or thromboxane-driven pathways, possibly reflecting differential receptor crosstalk or downstream kinase engagement. Circulating physiological concentrations of Ang-(1–7) are generally reported in the picomolar range (approximately 10–50 pM), which is substantially lower than the nanomolar concentrations used in the present experiments. Therefore, the concentrations applied in this study should be interpreted as pharmacological concentrations commonly required in isolated vascular preparations to overcome peptide degradation, diffusion barriers within the tissue, and reduced receptor accessibility, rather than as direct reflections of physiological circulating levels [18,19,27,29,32,33,34]. Micromolar concentrations are frequently required in isolated tissue experiments to overcome peptide degradation, limited receptor density, and competing vasoactive influences. While these concentrations sometimes are pharmacological rather than physiological, they enable characterization of K^+^ channel-dependent mechanisms and receptor-linked signaling pathways that may become relevant under conditions of enhanced local Ang-(1–7) activity or therapeutic administration [17,19,27,33]. Furthermore, tissue-level concentrations within the vascular wall may differ substantially from circulating plasma levels, particularly in pathological states characterized by local RAAS activation.

From a translational perspective, excessive venoconstriction contributes to elevated cardiac preload and venous congestion in cardiovascular disorders such as hypertension and heart failure. Therefore, modulation of venous tone through activation of the ACE2/Ang-(1–7)/Mas axis may represent a complementary strategy to limit pathological increases in venous return. In heart failure, where increased venous tone augments mean circulatory filling pressure and worsens congestion, targeted enhancement of Ang-(1–7) signaling could theoretically improve preload conditions without directly affecting arterial resistance. However, clinical translation of Ang-(1–7)-based therapies remains limited by their short half-life, low bioavailability, and potential interactions with other RAAS-modulating agents [14,35]. Consequently, development of stable Ang-(1–7) analogs, non-peptide Mas receptor agonists, or ACE2 activators represents an important area of ongoing pharmacological research.

Finally, the present study has limitations. From a physiological perspective, modulation of venous tone may influence preload, stressed blood volume, and mean circulatory filling pressure, thereby potentially affecting cardiac output and systemic congestion. Although in vivo validation was beyond the scope of this study, these findings provide a mechanistic basis for future investigation of venous-targeted RAAS modulation. Targeting the ACE2/Ang-(1–7)/Mas axis in venous smooth muscle may offer a novel strategy to reduce pathological venous constriction, lower cardiac preload, and mitigate congestion in hypertension or heart failure. Experiments were limited to isolated rat inferior vena cava preparations, and in vivo hemodynamic validation was not performed. Nevertheless, isolated tissue experiments allow direct evaluation of vascular smooth muscle mechanisms independent of neural, hormonal, and systemic regulatory influences. In addition, TEA was used as a broad pharmacological probe to assess the overall involvement of potassium channels in the Ang-(1–7) response. Because TEA is a non-selective potassium channel blocker, the present study cannot identify the specific potassium channel subtype involved. Future investigations incorporating receptor-selective antagonists, subtype-specific potassium channel inhibitors, and molecular signaling approaches will be necessary to further clarify the intracellular pathways involved. Moreover, integration of these mechanistic studies with in vivo hemodynamic assessment will be required to fully define the physiological and translational relevance of Ang-(1–7) in venous regulation. In summary, Ang-(1–7) exerts significant agonist-dependent anti-contractile effects in the rat inferior vena cava that are associated with activation of TEA-sensitive potassium channels in venous smooth muscle. These findings broaden the functional scope of the ACE2/Ang-(1–7)/Mas axis to include direct modulation of venous capacitance and preload regulation, thereby expanding its recognized cardiovascular protective profile beyond arterial resistance vessels. The present work provides mechanistic groundwork for future translational investigations into venous-targeted RAAS modulation and supports the concept that the ACE2/Ang-(1–7)/Mas axis contributes to integrated vascular regulation across both arterial and venous compartments.

## 5. Conclusions

Ang-(1–7) exerts significant anti-contractile and vasodilatory effects in the rat inferior vena cava, predominantly through activation of TEA-sensitive K^+^ channels in vascular smooth muscle. The marked attenuation of its effects by TEA, together with the lack of involvement of NO- or prostaglandin-dependent pathways under our experimental conditions, supports a primarily smooth muscle-centered hyperpolarization mechanism. These effects attenuate Ang II-induced venoconstriction and demonstrate that the ACE2/Ang-(1–7)/Mas receptor axis is functionally active in a major venous capacitance vessel. By extending the mechanistic understanding of Ang-(1–7) from arterial resistance vessels to venous smooth muscle, the present study highlights a previously underexplored component of RAAS-mediated vascular regulation. Importantly, this work provides direct experimental evidence that the protective ACE2/Ang-(1–7)/Mas axis modulates venous capacitance function rather than being inferred solely from arterial data. While the present work proves the in vitro vasorelaxant effect of externally administered Ang (1–7) in venous tissue, the real physiological and pathological significance of such a relaxation should be tested and further proven in the future by in vivo experiments. Also, the cellular mechanism has to be cleared in more detail.

## Figures and Tables

**Table 1:** Maximal relaxation responses (E_max_, %) induced by Ang-(1–7) in inferior vena cava rings pre-constricted with different agonists.

Preconstrictor (Agonists)	Ang-(1–7) Relaxation Response (E_max_, %)
PE	E_max_ = 35.00 ± 4.50
ET-1	E_max_ = 15.00 ± 2.80 ***
U46619	E_max_ = 12.00 ± 2.60 ***

Values represent maximal relaxation responses (E_max_) obtained at the highest Ang-(1–7) concentration (10^−5^ M) following cumulative concentration–response curves (10^−9^–10^−5^ M). Relaxation responses are expressed as a percentage (%) of the initial precontraction induced by each agonist. Data are presented as mean ± SEM (*n* = 8). Statistical significance was evaluated using two-way analysis of variance (ANOVA) followed by Bonferroni’s post hoc test. *** *p* < 0.001 versus PE-precontracted rings.

## Data Availability

The data presented in this study are available from the corresponding author upon request. The data are not publicly available due to ethical and institutional restrictions related to animal research and data management policies.

## Abbreviations

The following abbreviations are used in this manuscript:

ACE	Angiotensin Convert Enzyme
Ang II	Angiotensin 2
Ang-(1–7)	Angiotensin 1–7
IVC	Inferior Vena Cava
ET-1	Endothelin-1
PE	Phenylephrine
RAAS	Renin-Angiotensin-Aldosterone System
